# Serious Games for Mental Health: Are They Accessible, Feasible, and Effective? A Systematic Review and Meta-analysis

**DOI:** 10.3389/fpsyt.2016.00209

**Published:** 2017-01-18

**Authors:** Ho Ming Lau, Johannes H. Smit, Theresa M. Fleming, Heleen Riper

**Affiliations:** ^1^Department of Psychiatry, The EMGO Institute for Health and Care Research, VU University Medical Center Amsterdam, GGZ inGeest, Amsterdam, Netherlands; ^2^Department of Psychological Medicine, University of Auckland, Auckland, New Zealand; ^3^Department of Clinical, Neuro and Developmental Psychology, Section of Clinical Psychology, VU University Amsterdam, Amsterdam, Netherlands

**Keywords:** serious games, gamification, game-based intervention, depression, anxiety, post-traumatic stress disorder, alcohol, attention

## Abstract

**Introduction:**

The development and use of serious games for mental health disorders are on the rise. Yet, little is known about the impact of these games on clinical mental health symptoms. We conducted a systematic review and meta-analysis of randomized controlled trials that evaluated the effectiveness of serious games on symptoms of mental disorder.

**Method:**

We conducted a systematic search in the PubMed, PsycINFO, and Embase databases, using mental health and serious games-related keywords. Ten studies met the inclusion criteria and were included in the review, and nine studies were included in the meta-analysis.

**Results:**

All of the serious games were provided *via* personal computer, mostly on CD-ROM without the need for an internet connection. The studies targeted age groups ranging from 7 to 80 years old. The serious games focused on symptoms of depression (*n* = 2), post-traumatic stress disorder (*n* = 2), autism spectrum disorder (*n* = 2), attention deficit hyperactivity disorder (*n* = 1), cognitive functioning (*n* = 2), and alcohol use disorder (*n* = 1). The studies used goal-oriented (*n* = 4) and cognitive training games (*n* = 6). A total of 674 participants were included in the meta-analysis (380 in experimental and 294 in control groups). A meta-analysis of 9 studies comprising 10 comparisons, using a random effects model, showed a moderate effect on improvement of symptoms [*g* = 0.55 (95% confidence interval 0.28–0.83); *P* < 0.001], favoring serious games over no intervention controls.

**Discussion/conclusion:**

Though the number of comparisons in the meta-analysis was small, these findings suggest that serious gaming interventions may be effective for reducing disorder-related symptoms. More studies are needed in order to attain deeper knowledge of the efficacy for specific mental disorders and the longer term effects of this new type of treatment for mental disorders.

## Introduction

Serious games are “games that do not have entertainment, enjoyment or fun as their primary purpose” (1). Primary purposes of serious games can be, but are not limited to, education, training, human resource management, and health improvement (2). The term “serious games” was introduced more than a decade ago (3). Since then, the development and use of serious games have grown (4). A Google search for the term “serious games” shows approximately 3.4 million entries in 2016 (search conducted by us on August 8, 2016), compared to some 1.1 million entries that was found using the same search string in 2007 (5). The definition of serious games is, however, still evolving. Various definitions of serious games can be found in the literature (1, 3, 6–9). Some definitions focus on the technological aspect of the games (3, 8), while others focus more on training (7) or educational purposes (6). In the current study, we used the following definition of serious games: games that are designed to educate, train, or change behavior as they entertain players (10). Serious games can be non-digital (11); however, most serious games in the peer-reviewed literature are delivered online or *via* stand-alone computer technology.

Serious games have found their way into health care (12, 13) as shown by the increase in releases in this sector from 4.7% in 2002 to 8.2% in 2011 of the total serious games market (4). For example, the serious game *Re-Mission* was developed in order to actively involve young people with cancer in their own treatment by educating them on cancer and its treatment (14). In recent years, the potential use of serious games in mental health care has also been explored. For example, a web-based social network electronic game designed to enhance mental health literacy in young people was developed and evaluated in a pre- and posttest design. This gaming approach was found to be effective for this purpose (*d* = 0.65) (15). Also, a review of the potential of using games to improve mental health professionals’ knowledge as a teaching strategy found that those allocated to educational games performed considerably better on a mental health nursing test than those health professionals who were not (16). In addition, the deployment and design of serious games for psychotherapy has recently been studied (17, 18). In a review of literature on the use of video games in psychotherapy, Ceranoglu (17) concluded that games will be likely to be used in psychotherapy as therapists gain familiarity with gaming equipment. Further, games have been developed to treat impulse-related conditions such as eating disorders (19, 20). Initial results of an evaluation study showed that patients with eating disorders feel comfortable using a serious game in treatment (20).

Other forms of digital interventions for mental disorders already exist and have been studied more extensively. Internet and computerized interventions are found to be effective for the prevention and treatment of adult common mental disorders, such as depression, anxiety, and alcohol use disorders (AUDs) (21–24). There is also evidence, albeit limited, that computerized cognitive behavioral therapy (CBT) is effective for the treatment of anxiety and depressive symptoms among young people (25–27).

Digital serious games may enrich the array of digital interventions due to their specific characteristics such as the provision of an alternative world in which learning and exploration is encouraged (2, 28). Serious games may make learning more meaningful, engaging, and challenging than traditional teaching by using the interactive, visual, and immersive characteristics available in video games (29). They may help children and adults alike to develop simpler solutions and become more creative in solving problems (30). Looking at these characteristics and benefits of video games, it seems attractive that these are being explored for their potential use in the prevention and treatment of mental disorders (31). Whether it is using games to serve a serious purpose or gamify a serious purpose, the goal is to help individuals reduce mental health complaints or improve their mental wellbeing. But how do these mental health games perform on improving mental health? Are there evidence-based serious games for the treatment or prevention of mental health symptoms?

To date, few serious games have been tested and reported in the scientific literature. A pilot study was conducted to investigate the effectiveness of a brain–computer interface in the treatment of attention deficit hyperactivity disorder (ADHD) (32). The results showed improvement in inattentive symptoms and hyperactive–impulsive symptoms after playing on the attention training game system. Furthermore, a case study has been conducted using a serious game in the treatment of specific phobia (33), indicating that serious games are helpful in reducing fear and avoidance. Results of these randomized controlled trials (RCTs) show that serious games have the potential to be used as a whole or part of treatment for mental health disorders (34, 35). Recent reviews offer a broad view on serious games or game-based digital interventions within the mental health field. Li et al. (36) have focused on game-based digital interventions for depression, including serious games, but also simulations without game elements such as virtual reality exposure therapy (VRET). VRET is exposure therapy that makes use of virtual reality (VR) to simulate a real-world situation in order to treat a specific phobia. Support for the effectiveness of game-based interventions for depression was found. In another review of serious games and mental health conducted by Van der Krieke et al. (37), the scope also comprised simulations without game elements (38, 39), VRET (40, 41), and interactive computerized interventions (42). Games and simulations are, however, different conceptual entities (43). Simulations do not necessarily contain a competitive or conflict element, in contrast to games where users try to win or cope with certain problems bounded by rules. Although some video games are based on simulations, simulations without game elements were not considered as (serious) games for this study. VRET, simulations, and interactive computerized interventions do not necessarily contain elements that make a game *a game* and thus should not be categorized as serious games (43). Also, since the available reviews (36, 37) included studies that are not all RCTs, questions about effectiveness could not be answered optimally. Thus while a few reviews (36, 37) have been conducted on the potential impact of serious games for common mental disorders, none of these are robust. Therefore, a contemporary update is called for, given the rapidly evolving field; and an evaluation of trials to date by means of a meta-analytic review is still lacking.

The current study aims to systematically evaluate studies that have assessed the effectiveness of serious games in treatment outcomes for mental disorder-related symptoms by means of a systematic review including a meta-analysis.

## Materials and Methods

“The Preferred Reporting Items for Systematic Reviews and Meta-Analyses (PRISMA)” statement (44) was used as guideline to conduct this study.

### Search and Study Selection

A literature search of the PubMed, PsycINFO, and Embase databases was conducted. The search string was a combination of serious games-related terms, such as “serious games,” “game-based,” “videogames,” “computer-assisted therapy,” “virtual reality intervention,” “gamification,” “gaming simulation,” and mental health-related terms, such as “mental health,” “depression,” “anxiety,” “problem drinking,” “schizophrenia,” and “obsessive-compulsive disorder.” Duplicate items were removed from the records that were identified through the literature search. The remaining items were screened on basis of title, abstract, and keywords by two independent raters (Ho Ming Lau and Ka Wai Ma). Items were included if the following inclusion criteria were met (a) the intervention used a digital game delivered on any technical platform including personal computers (PCs), consoles, cell phones, and handheld devices, which means that non-digital games were excluded; (b) the intervention targeted mental disorders such as those mentioned above; and (c) the study conducted an RCT. For the purpose of our study, simulations, VR interventions, and interactive programs without game elements were not considered serious games and were therefore excluded. The remaining records were assessed for eligibility by two independent raters (Ho Ming Lau and Jan Smit Jr.). Differences in ratings were resolved by discussion till a consensus was reached. If no consensus was reached, the coauthors of this paper were consulted to make the final decision.

### Data Extraction and Synthesis

A data extraction sheet was developed and pretested on two studies. The variables that were extracted from the articles were divided into two categories: (1) participant and study characteristics and (2) game characteristics.

Participant and study characteristics included variables, such as target group, recruitment, treatment type (single- or multicomponent intervention), primary outcome measures, how and how much guidance during intervention was given, setting of intervention, study conditions, attrition, and results.

Game characteristics comprised variables, such as title of the game used in the study, serious game type, game genre, and purpose of the game.

### Quality Assessment

The validity of the included studies was assessed using the Risk of Bias Assessment tool of the Cochrane Collaboration (45). We used the following six criteria: random sequence generation, allocation concealment, blinding of participants and personnel, blinding of outcome assessors, incomplete outcome data, and selective outcome reporting. Each type of risk bias was rated low, high, or unclear.

### Meta-analysis

Analysis of the data was done using the program Comprehensive Meta-Analysis (CMA) (46). Cohen’s *d* is an effect size that can be used to indicate the standardized difference between the two means divided by the pooled standard deviation at posttest. Hedges’ *g*, a variation of Cohen’s *d*, can be used to correct for potential bias as a result of small sample sizes. In this study, Hedges’ *g* was applied due to a number of studies with small sample sizes. An effect size of 0.2 indicates a small effect, 0.5 indicates a moderate effect, and 0.8 to infinity indicates a large effect (47). For studies that did not provide means or standard deviations, statistics such as *F* value or *t* value were used to calculate the effect sizes according to formulae of CMA. It should be noted that one study conducted two experiments (48). Both experiments were included as separate comparisons in the meta-analysis. The random effects model was used to calculate the mean effect sizes, as we expected heterogeneity among the included studies. It assumes that the calculated effect sizes differ because of true variation in effect size from one study to the next and not only of the random error within studies. The *Q*-statistic was calculated to assess the presence versus absence of heterogeneity, but only significance was reported. The *I*^2^-statistic was also calculated to test the homogeneity of effect sizes. A value of 0% indicates no observed heterogeneity, while larger values indicate increasing heterogeneity, with 25% as low, 50% as moderate, and 75% as high (49). Furthermore, we estimated 95% confidence intervals (CIs) around *I*^2^ (50) using the non-central chi square-based approach within the Deducer GUI (51) of R (52).

The numbers needed to treat (NNTs) were calculated, using the formulae provided by Kraemer and Kupfer (53). By calculating the NNT, we obtained an estimation of the number of patients who need to be treated in order to have one who would benefit. The lower the NNT, the more effective the treatment is.

In studies where multiple primary outcomes were used, the effect sizes were averaged to produce a single summary effect size for use in the meta-analysis (54). Data were extracted by two independent reviewers (Ho Ming Lau and Jan Smit Jr.). Differences in extractions were resolved by consulting the coauthors.

Furthermore, two subgroup analyses were conducted according to the random effects model comparing (1) younger (≤18) versus older (>18) participants and (2) clinical versus non-clinical participants. We conducted additional subgroup analyses for the studies on the disorders, such as autism spectrum disorder (ASD), cognitive functioning, and post-traumatic stress disorder (PTSD) separately.

We examined the funnel plots visually in order to detect possible publication bias. A funnel plot is a simple scatterplot of the intervention effect against a measure of each study’s size. A symmetrical inverted appearance of the funnel plot indicates low publication bias, whereas an asymmetrical funnel plot indicates potential publication bias which may lead to an overestimation of the intervention effect (55). To verify an unbiased estimate of the pooled effect size, the Duval and Tweedie trim-and-fill analysis was performed (56). Moreover, Egger’s linear regression method was applied on the intercept to quantify the possible publication bias captured by the funnel plot and its significance.

## Results

### Study Selection

**Figure 1:**
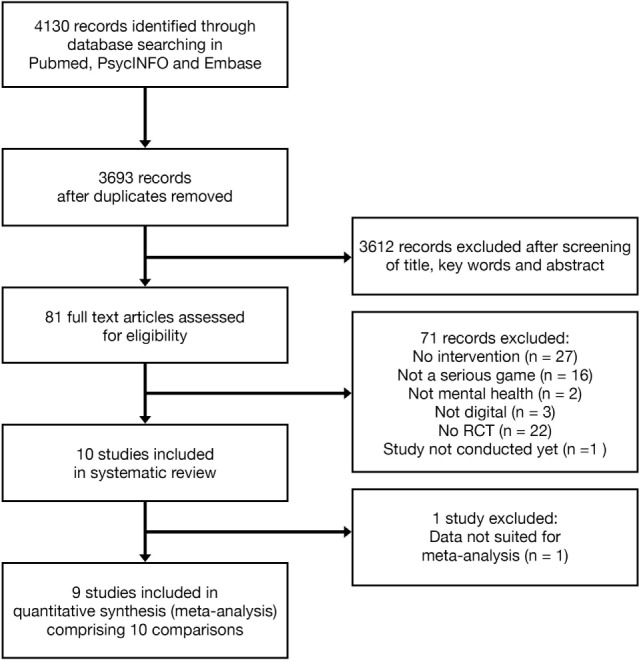
**Flowchart of study inclusion**.

The PubMed, PsycINFO, and Embase search returned 4,130 items. After removal of duplicates, 3,693 records remained. Initial screening of title and abstract excluded a further 3,612 articles, leaving 81 items. After applying the exclusion criteria, 10 studies were included in the review and 9 in the meta-analysis. See Figure 1 for a flow chart of the study selection.

### Results of the Review

We start by reviewing some characteristics of the serious games at stake, including their game design features per disorder (Table 1). We then present the results of our meta-analysis on the impact of these games on psychiatric-related disorders.

**Table 1:** **Characteristics of the randomized controlled trials that are included in the review**.

Reference, country	Target group	Recruitment	Treatment type	Primary outcome measures	Guidance (on game component)	Setting	Study conditions	*n* (% male)	pt and fu assessments	Study attrition (%)	Risk of bias
Ballesteros et al. (58), Sweden	Healthy elderly 57–80 years	Flyers, word of mouth, community centers	Single game	Speed of processing, attention, executive control, spatial working memory, episodic memory, and subjective wellbeing	No information	Research laboratory	1. SG 2. No intervention	1. 20 2. 20 (40%)	pt: 10–12 wk	pt: 25	1. n.i. 2. n.i. 3. n.i. 4. n.i. 5. Yes 6. No

Beaumont and Sofronoff (35), Australia	Children 7–12 years, ASD, WISC-III IQ score ≥85; DSM-IV-TR Asperger disorder	Newspaper, newsletter and letters to clients	SG with add-on group sessions	SSQ, ERSSQ, emotion recognition and emotion management	Virtual guide in game	Educational institution	1. SG 2. WL	1. 26 2. 23 (90%)	pt: 6 wk; fu: 5 mth	pt: unclear; fu: 6.5	1. n.i. 2. n.i. 3. n.i. 4. No 5. Yes 6. No

Dovis et al. (59), Netherlands	Children 8–12 years, DSM-IV-TR ADHD	Mental health-care centers	Single game	Stop task, Stroop, CBTT, Digit span, TMT, Raven colored progressive matrices, DBDRS, BRIEF, SPSRQ-C, PedsQL, HSQ	Instructions by researcher, weekly call by coach	Home	1. SG (fa) 2. SG (pa) 3. Placebo (ac)	1. 31 2. 28 3. 30 (80%)	pt: 6–7 wk; fu: 3 mth	pt: 3.4; fu: 9	1. No 2. No 3. No 4. No 5. No 6. No

Fleming et al. (34), New Zealand	Adolescents 13–16 years, CDRS-R depressive disorder	Schools, educational programs	Single game	CDRS-R	Minimal supervision by ESP, virtual guide in game	Educational institution	1. SG 2. WL	1. 20 2. 12 (56%)	pt: 5 wk; fu: 10 wk	pt: 3; fu: 16	1. No 2. Yes 3. Yes 4. Yes 5. No 6. No

Holmes et al. (64), UK	Adults 18–47 years	Unclear	Single game	Number of flashbacks, Impact of Event Scale	Unclear	Research laboratory	1. SG 2. No intervention	1. 20 2. 20 (55%)	pt: 1 wk	pt: unclear	1. n.i. 2. n.i. 3. n.i. 4. n.i. 5. No 6. No

Holmes et al. (48), UK	Non-clinical adults; Exp. 1: 18–60 years; Exp. 2: 18–57 years	Online ads and community	Single game	Number of flashbacks	No information	Research laboratory	1. SG 2. No intervention (CG) 3. Pub quiz	Exp.1: 1. 20 2. 20 3. 20 (50%)	pt: 1 wk	pt: unclear	1. n.i. 2. n.i. 3. n.i. 4. n.i. 5. No 6. No
								Exp.2: 1. 26 2. 26 3. 26 (46%)			

Merry et al. (60), New Zealand	Adolescents 12–19 years, clinically significant depression	Primary health-care sites	Single game	CDRS-R	Virtual guide in game	Health-care center	1. SG 2. TAU	1. 94 2. 93 (34%)	pt: 2 mth; fu: 5 mth	pt: 9; fu: 10	1. No 2. No 3. No 4. No 5. Yes 6. No

Rezaiyan et al. (61), Iran	Educable mentally challenged children	24-h care centers	Single game	Toulouse Pierson Scale	No information	Unclear	1. SG 2. No intervention	1. 30 2. 30 (100%)	pt: immediately after intervention; fu: 5 wk	pt: unclear; fu: unclear	1. n.i. 2. n.i. 3. n.i. 4. n.i. 5. No 6. No

Tanaka et al. (63), USA	Children to young adults, DSM-IV ASD	Presentations at schools and parent organizations, existing relationships with families	Single game	Face subtests, object subtests	Self-paced, not directly supervised; Games suggestions by parents based on compliance	Home	1. SG 2. WL	1. 42 2. 37 (79%)	pt: 19 wk (average)	pt: unclear	1. No 2. n.i. 3. n.i. 4. n.i. 5. Yes 6. No

Verduin et al. (62), USA	Male adult veterans 45–57 years, DSM-IV alcohol abuse or dependence	Veteran’s Administration Medical Center	SG adjunct to TAU	Relapse, OCDS, AUQ, TSSE-RP	No information	Health-care center	1. SG 2. Slides	1. 19 2. 22 (100%)	pt: 12 wk; fu: 16 wk	pt: unclear; fu: unclear	1. No 2. n.i. 3. n.i. 4. n.i. 5. No 6. No

*ac, active control; ADHD, attention deficit hyperactivity disorder; ASD, autism spectrum disorder; AUQ, Alcohol Urge Questionnaire; BRIEF, Behavior Rating Inventory of Executive Function Questionnaire; CBTT, Corsi block tapping task; CDRS-R, Child Depression Rating Scale Revised; CG, control group; DBDRS, Disruptive Behavior Disorder Rating Scale; DSM-IV, Diagnostic and Statistical Manual of Mental Disorders, fourth edition; DSM-IV-TR, Diagnostic and Statistical Manual of Mental Disorders, fourth edition, text revision; ERSSQ, Emotion Regulation and Social Skills Questionnaire; ESP, Educational Service Provider; Exp., experiment; fa, full-active condition; fu, follow-up; HSQ, The Home Situations Questionnaire; mth, month (after start intervention); n.i., no information; PedsQL, Pediatric Quality of Life Inventory (parent and child versions); OCDS, Obsessive Compulsive Drinking Scale; pa, partially active condition; pt, posttreatment; SG, serious games condition; SPSRQ-C, Sensitivity to Punishment and Sensitivity to Reward Questionnaire for children; SSQ, Social Skills Questionnaire (parent and teacher forms); TAU, treatment as usual; TMT, Trail Making Test; TSSE-RP, Task-Specific Self-Efficacy for Relapse Prevention Questionnaire; WISC-III, Wechsler Intelligence Scale for Children, third edition; wk, week (after start intervention); WL, waitlist*.

#### Game Characteristics

An overview of the game characteristics can be found in Table 2. Three types of serious games in terms of design processes can be identified, namely, designed, purpose-shifted, and modified games (4). Designed serious games are games that are designed with a “serious” purpose from the beginning. Purpose-shifted serious games are games that were not designed as a serious game but are being used for a serious purpose. An example is the use of the game Tetris as part of an intervention to reduce the number flashbacks in PTSD research (48). Modified serious games are similar to purpose-shifted ones, but while purpose-shifted games are left intact, modified ones can differ from the original in terms of gameplay and characters. An example is modifying the engine of the first-person shooter game “Unreal” into a firemen training program (57). All three types (designed, purpose-shifted, and modified) of serious games were of interest for the current study. However, we found no modified games in our study. Eight studies used serious games that were designed as such (34, 35, 58–63). Two studies used an existing entertainment game for a serious purpose (purpose-shifted) (48, 64).

**Table 2:** **Game characteristics of the randomized controlled trials that are included in the review**.

Reference, country	Title	Serious game type	Serious game genre	Serious game purpose
Ballesteros et al. (58), Sweden	Games selected from Lumosity (cognitive training platform)	Designed	Cognition/brain training	Training (physical/emotional/cognition/skills)
Beaumont and Sofronoff (35), Australia	Junior Detective Program	Designed	Goal-oriented and problem-solving	Psychoeducation and training (physical/emotional/cognition/skills)
Dovis et al. (59), Netherlands	Braingame Brian	Designed	Cognition/brain training	Training (physical/emotional/cognition/skills)
Fleming et al. (34), New Zealand	SPARX	Designed	Goal-oriented and problem-solving	Psychoeducation and training (physical/emotional/cognition/skills)
Holmes et al. (64), UK	Tetris	Purpose-shifted	Cognition/brain training	Training (physical/emotional/cognition/skills)
Holmes et al. (48), UK	Tetris	Purpose-shifted	Cognition/brain training	Training (physical/emotional/cognition/skills)
Merry et al. (60), New Zealand	SPARX	Designed	Goal-oriented and problem-solving	Psychoeducation and training (physical/emotional/cognition/skills)
Rezaiyan et al. (61), Iran	“Path-finding game”	Designed	Cognition/brain training	Training (physical/emotional/cognition/skills)
Tanaka et al. (63), USA	Let’s Face It!	Designed	Cognition/brain training	Training (physical/emotional/cognition/skills)
Verduin et al. (62), USA	Guardian Angel	Designed	Goal-oriented	Training (physical/emotional/cognition/skills)

We divided the variable game genre into goal-oriented, problem-solving, cognition training, and so-called exergames. Goal-oriented games focus on tasks and the end results of those tasks. Problem-solving games challenge players to find solutions for problems. Cognition training games train the players’ working memories by a series of similar brief challenges that usually have to be tackled within time constraints. Exergames are games that combine physical exercises with game elements. No exergames were identified in the current study. Six studies used serious games in the cognition training genre (48, 58, 59, 61, 63, 65). Three studies used serious games that can be categorized in two genres, namely, goal-oriented and problem-solving (34, 35, 60). One study used a serious game that was goal-oriented only (62).

All the studies used serious games for training purposes. Three of these 10 studies also had psychoeducation as a purpose (34, 35, 60).

#### Depression

Two RCTs targeted depression, both with the same serious game SPARX (34, 60), aimed at adolescents (aged from 12 to 19 years). In both studies, this goal-oriented and problem-solving game was used in order to reduce depression-related symptoms. This game is based on CBT (66). The version of *SPARX* used in the studies can be played on a PC without the need for an internet connection (however, an online version is now available at https://www.sparx.org.nz/); it can be used free of charge by collaborators in New Zealand only currently. In this game, the player controls a personalized character who has to restore the balance in a fantasy world, for instance, by solving problems and shooting negative thoughts, all components of CBT. The player is guided by a virtual character who speaks about dealing with depression and gives instructions and objectives for the seven levels in the game. Each level (or module) has a duration of approximately 30 min. The game was played under minimal supervision from educational service providers in Fleming et al. (34). The Children’s Depression Rating Scale Revised (67) was used as primary outcome measure for both studies. In Merry et al. (60), SPARX was played on primary health-care or school guidance center/health locations.

#### Post-traumatic Stress Disorder

Post-traumatic stress disorder was the focus in two serious game studies (48, 64). A study by Holmes et al. (48) was conducted in an attempt to deal with the limitations in an earlier study (64). *Tetris* was used in both studies in order to reduce PTSD-related symptoms. The theory of using *Tetris* is based on findings in cognitive science and neurobiology of memory. Flashbacks of traumatic events are assumed to consist of sensory-perceptual and visuospatial mental images. When visuospatial tasks are performed after a traumatic event and also within the time window of memory consolidation, competition for the same resources will occur, causing interference with and reduction of flashbacks. *Tetris* (68) was purpose-shifted, since it was originally developed for entertainment. Nowadays, *Tetris* is available on different devices and platforms (e.g., mobile, tablet, and game consoles). In both studies, the game was played on a PC in the research lab at the university. No internet connection was needed to play *Tetris*. In this game, a random sequence of geometric shapes consisting of four square blocks each (Tetriminos) fall down the playing field. The player has to try to make horizontal lines without gaps with the Tetriminos. When a full line is created, this line of blocks will disappear and the blocks on top of the line will fall. When the blocks reach the top of the playing field and thus no new Tetriminos are able to enter, the game ends. Within both studies by Holmes et al. (48, 64), this game was played for 10 min with little instruction needed, after watching a film containing traumatic scenes.

#### Autism Spectrum Disorder

Two studies used serious games to address symptoms of ASD (35, 63). Beaumont and Sofronoff (35) and Tanaka et al. (63) had facial expression as common primary outcome measure. The games are based on the theory of enhancement of emotional understanding and social skills (35) through training. The goal-oriented and problem-solving game *Junior Detective Training Program* (*JDTP*) was used in order to reduce social skills impairment (35). *JDTP* can be played on a PC without the need for an internet connection. In this game, the player is a junior detective in the year 2030 who is specialized in decoding suspects’ thoughts and feelings. The player plays three levels with different missions including decoding suspects’ feelings through facial expressions and body postures, deciphering cartoon character’s feelings in different situations from non-verbal and environmental clues, dealing with bullying, and playing with others. After completing the three levels, the player graduates from the “Detective Academy.” The games group was asked to play for the first hour in the first two sessions and 45 min per session in the third and fourth session. The seven sessions in total also comprised training time for parents. As primary outcome measures, one study used emotion recognition (facial expression and body posture) and emotion management (*Dylan is being teased*, a coping with bullying test and *James and the Maths Test*, a coping with anxiety test) (35).

The games of Tanaka et al. (63) are based on the theory of enhancement of recognition skills (63). The cognition training game *Let’s Face It!* was used in order to reduce ASD-related symptoms such as poor facial recognition skills (63). *Let’s Face It!* is available to the public and can be downloaded free of charge from the website of the University of Victoria (http://web.uvic.ca/~letsface/letsfaceit/); however, supervision of the player is recommended. The game can be played on the PC (or Mac) without internet connection after downloading. In *Let’s Face It!*, the player plays face and object recognition games that target face processing skills, e.g., matching faces and connecting faces of the same identity. Participants were instructed to play the games for at least 100 min/week at home until intervention time reached 20 h. The parents received advice from the researchers about which games their children should play based on the data collected on compliance and the child’s game play. *Let’s Face It!* used face subtests (face dimensions, immediate memory for faces, matching identity, masked features, expression, and parts/whole identity) and object subtests (house dimensions and immediate memory for cars) (63). Data collection was done pre- and post-intervention. The intervention had a duration of 19.1 weeks average (63).

#### Attention Deficit Hyperactivity Disorder

Symptoms related to ADHD were targeted in one study (59). The serious game is based on ADHD theories which argue that deficits in executive functioning are related to impulsivity, hyperactivity, and attention (69–77). The cognition training PC game *Braingame Brian* (78) was used in order to reduce ADHD-related symptoms. In the game, the participant plays as Brian, a young inventor who helps and befriends in-game characters by creating inventions. The games consisted of a working memory task, a cognitive flexibility task, and an inhibition task. The game was played for 25 sessions of 35–50 min each.

#### Cognitive Functioning

Two studies targeted cognitive functioning symptoms (58, 61), meaning limited attention capacity due to impairment (61) and age-related decline in cognitive performances including working memory, speed of processing, and cognitive control (58).

The serious games studied by Rezaiyan et al. (61) are based on the finding that playing video games based on internal motivation can be a source of increasing attention power (61). A cognition training computer games program that focused on path-finding (proceeding from easy to hard) was used in order to reduce cognitive decline symptoms (61). The cognition training games were played for 35 sessions of 20–30 min each in one study (61).

Ballesteros et al. (58) targeted children with cognitive decline symptoms, determined by the Toulouse Pierson Scale. The serious games are based on theories of neuroplasticity (58). Ballesteros et al. (58) pursued a reduction in cognitive decline symptoms by using the commercially available cognition training PC platform Lumosity (79). This game is also available on mobile devices. The serious games were played for 20 sessions of 1 h each (58).

#### Alcohol Use Disorder

Treatment of AUD symptoms was of interest in one study (62). The participants were recruited at a veterans’ outpatient medical center. The goal-oriented PC game *Guardian Angel* (80) is based on cognitive behavioral approaches (81). *Guardian Angel* was designed and used to reduce AUD-related symptoms. The participants played the game on a laptop at the medical center. In this game, the player acts as a “guardian angel” that needs to guide a character in early recovery of AUD to make daily decisions in support of recovery and continued abstinence. Players have to recognize and remove relapse risk factors. *Guardian Angel* emphasizes relapse prevention intervention techniques including identification of high-risk situations, drink-refusal skills, stimulus control, and craving-management techniques. The game was to be played during eight sessions over the course of 12 weeks. Participants in the game condition played 1 h per session, with the opportunity to play up to 8 h per session.

### Results of the Meta-analysis

#### Study Characteristics

The participants and study characteristics of the included studies are presented in Table 1. The 10 included studies were conducted in various geographical regions ranging from Europe [Sweden (58), the United Kingdom (48, 64), the Netherlands (59)], to Australia (35), New Zealand (34, 60), Asia [Iran (61)] and the United States (62, 63). A total of 674 participants were included in the meta-analysis (380 in experimental and 294 in control group). Total sample sizes ranged from 32 to 89 participants. Two studies targeted depression (34, 60). One study focused on ADHD (59). AUD was targeted in one study (62). PTSD was the subject of two studies (48, 64). Cognitive functioning was targeted in two studies (58, 61). ASD was the focus of two studies (35, 63). Four studies were aimed at children aged between 7 and 12 years (35, 59, 61, 63), two studies focused on teens to young adults (aged between 12 and 18), three studies were aimed at adults (18+) (34, 48, 60, 62, 64), and one study was aimed at older adults aged between 57 and 80 years (58). Six studies compared serious games to no intervention (34, 35, 58, 61, 63, 64); three studies compared them to active controls, such as training cognitive tasks, playing a quiz, and watching slides (48, 59, 62); and one study compared gaming to treatment as usual (TAU) (60).

Two studies used serious games in adjunct to TAU (35, 62). The serious games were played in different settings: three studies were conducted in a research laboratory (48, 58, 64), two at an educational institution (34, 35), two at home (59, 63), two at a health-care center (60), and one remained unclear (61).

The total number of comparisons that could be included in the analysis was *n* = 9. The study of Verduin et al. (62) on AUD did not provide amenable data for inclusion in the meta-analysis and is therefore excluded (Table 3).

**Table 3:** **Effects of serious games on reducing psychiatric disorder-related symptoms in comparison with control groups and two subgroup analyses**.

Serious games versus no intervention	Subgroup	*n* comp	*g*	95% CI	*I*^2^	95% CI^a^	*P*^b^	NNT
All studies		10	0.55	0.28–0.83*	58.53	1.31–34.15**		3.31
Excluded Merry et al. (60)		9	0.63	0.36–0.90*	42.90	0.00–23.77		2.91
One effect size per study (lowest and Merry et al. (60) excluded)		8	0.71	0.43–0.98*	29.61	0.00–18.40		2.60
One effect size per study (highest and Merry et al. (60) excluded)		8	0.56	0.30–0.81*	30.81	0.00–18.70		3.25
Attention deficit hyperactivity disorder		1	0.22	−0.40–0.83	0	n/a		8.06
Autism spectrum disorder		2	0.46	0.10–0.81	73.01	0.00–15.11		3.91
Cognitive functioning		2	0.79	0.36–1.21**	59.99	0.00–12.54		2.36
Depression		1	1.36	0.58–2.13**	0	n/a		1.51
Post-traumatic stress disorder		3	0.59	0.20–0.99**	0	0.00–7.84		3.09
Subgroup analyses								
Age	≤18	5	0.70	0.32–1.07	66.82	0.00–25.72**	0.61	2.63
	>18	4	0.53	0.07–0.99	0	0.00–7.31		3.42
Participant type	Clinical	4	0.59	0.18–0.99	64.35	0.00–21.04**	0.61	3.09
	Non-clinical	5	0.68	0.26–1.09	24.98	0.00–14.08		2.70

*CI, confidence interval; n comp, number of comparisons; NNT, number needed to treat; n/a, not available, could not be calculated due to the number of comparisons*.

*^a^The *P* values in this column indicate whether the *Q*-statistic is significant (*I*^2^-statistics do not include a test of significance)*.

*^b^The P values in this column indicate whether the difference between the effect sizes in the subgroups is significant*.

*P ≤ 0.001.

**P ≤ 0.05.

These studies evaluated the effectiveness of serious games across a broad range of mental disorders and outcomes. The common ground between the included studies is, however, the evaluation through RTCs of possible improvement to mental health symptoms using serious games. Though the studies varied in the use of outcome measures for behavior change, its construct may be measured validly using meta-analytic methods by taking an average of outcome measures of the same construct (82). Firstly, an overall meta-analysis was conducted, which means that we looked at whether participants in the serious game conditions improved over the control conditions. Secondly, a meta-analysis was conducted per disorder using a similar approach.

### Risk of Bias

**Figure 2:**
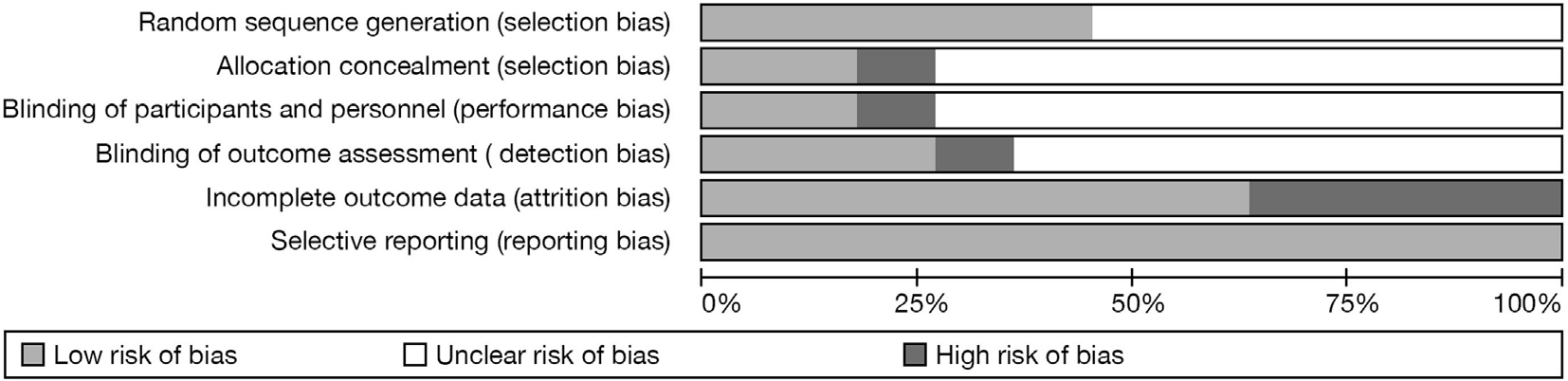
**Risk of bias graph**.

Figure 2 shows the results of the methodological assessment of the included studies in the meta-analysis. Five studies reported adequate random sequence generation (34, 59, 60, 62, 63). Two studies reported allocation to be concealed (59, 60). Two studies reported that both participants and personnel were blinded (59, 60). Three studies reported that research assistants were blinded for the outcome assessment (35, 59, 60). Four studies had a high likelihood of incomplete outcome data (35, 58, 60, 63). Several domains were rated unclear—the reason for this was incomplete reporting.

### Publication Bias

Visual inspection of the funnel plot indicated possible publication bias. Performance of the Duval and Tweedie trim-and-fill procedure also indicated possible publication bias. After adjustment for missing studies, the effect size for serious games interventions changed from *g* = 0.63 to 0.48 (95% CI 0.17–0.78; trimmed studies = 2). The Egger’s test did not indicate an asymmetrical funnel plot (*P* > 0.10).

### Meta-analyses Outcomes

**Figure 3:**
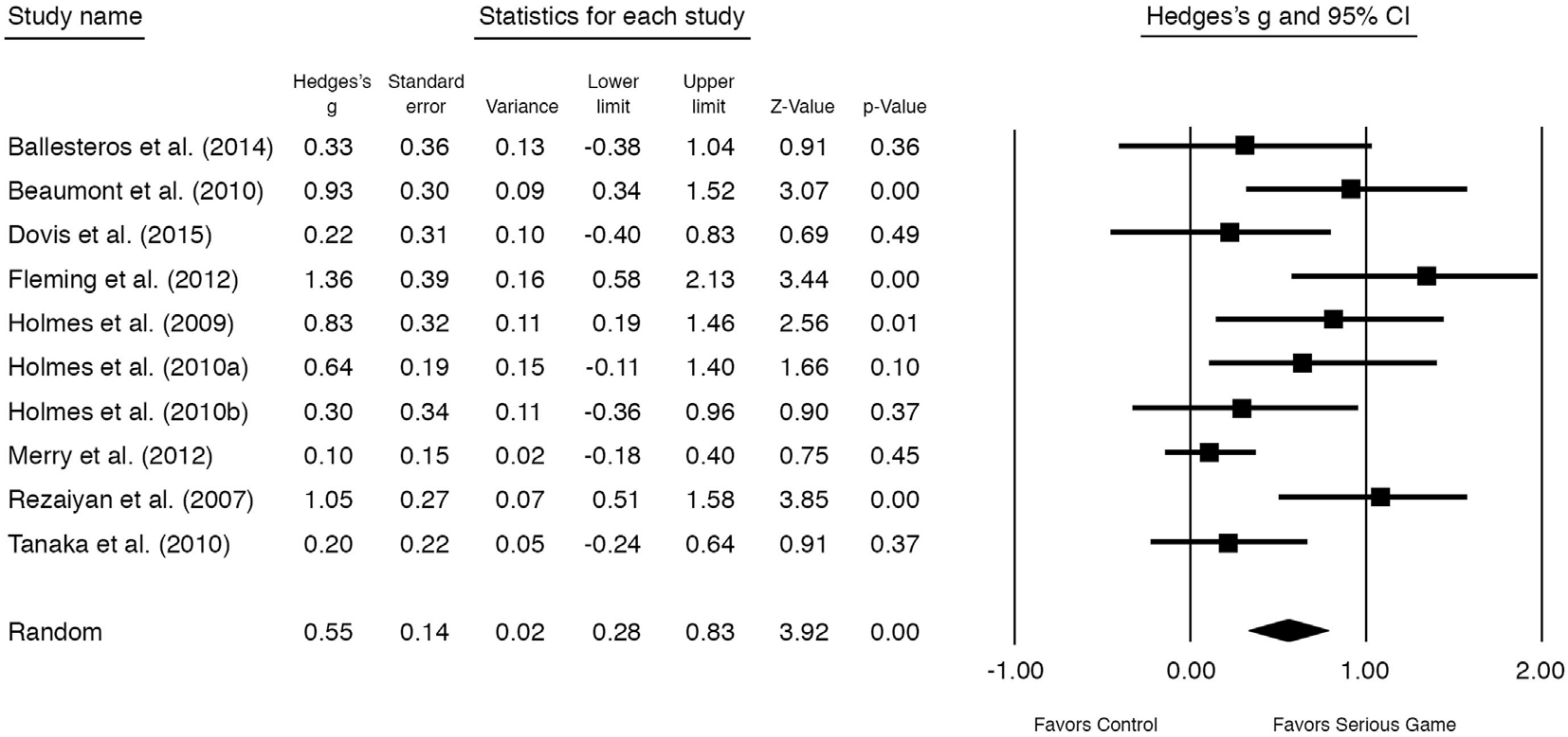
**Standardized effect sizes of serious gaming interventions for mental health compared with control group**.

**Figure 4:**
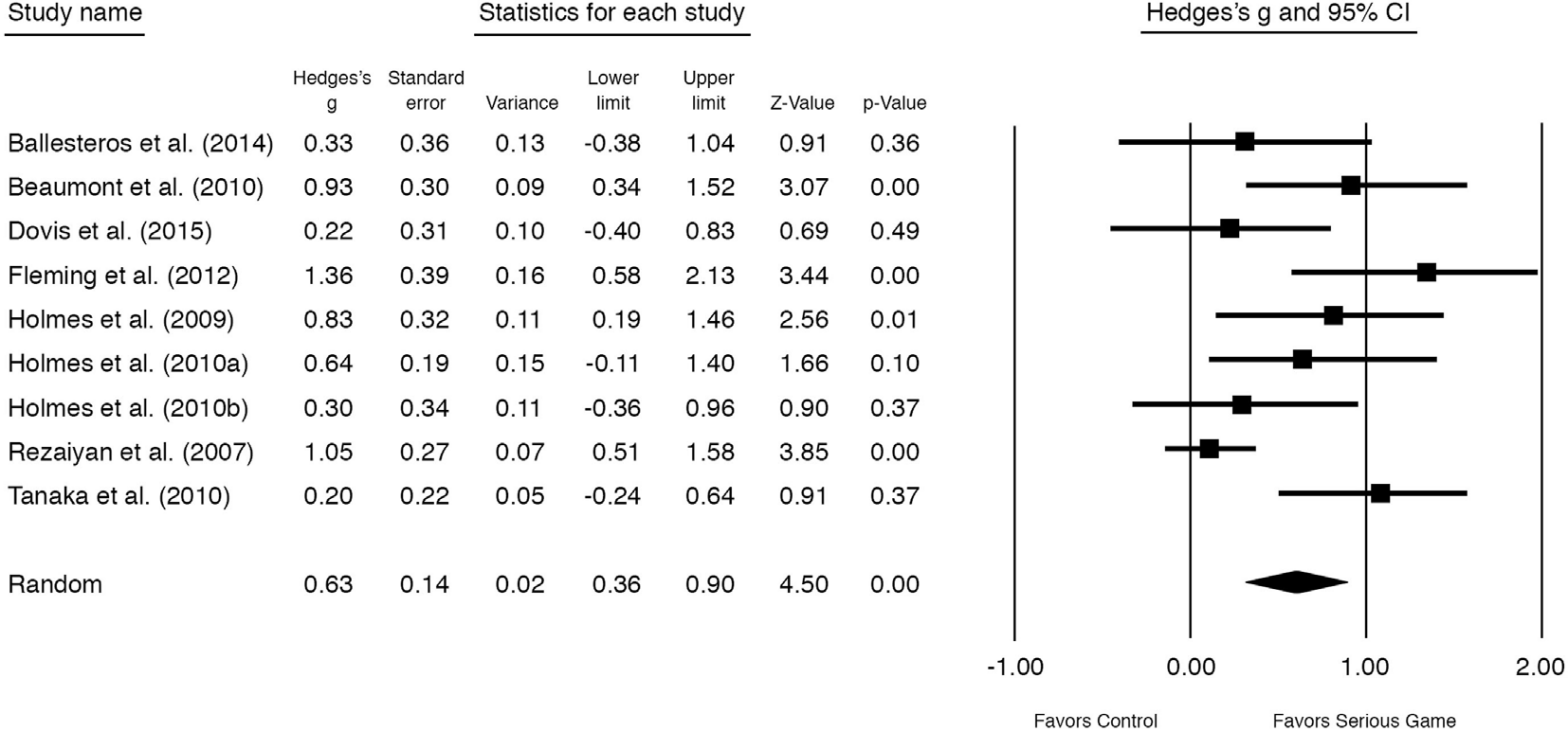
**Standardized effect sizes of serious gaming interventions for mental health compared with control group**. *Note*: study with treatment as usual as control group removed from analysis.

The overall outcome of the nine studies (*n* = 10 comparisons) showed a moderately significant effect size of *g* = 0.55 (95% CI 0.28–0.83, *P* < 0.001) for improvement on mental disorder symptoms at posttest (see Figure 3). Heterogeneity was substantial and significant (*I*^2^ = 58.53, 95% CI 1.31–34.15, *P* < 0.05) (Table 3). One study (60) had a control group that received TAU in contrast to the other studies (no treatment or waitlist). After excluding this study, a significant moderate almost similar effect size remained, *g* = 0.63 [95% CI 0.36–0.90, *P* < 0.001, NNT = 2.91] (Figure 4). Excluding the study with the highest (34) and lowest (63) effect size showed similar results (see Table 3).

We then conducted meta-analyses on psychiatric disorder-related symptoms for those where two or more studies were available (Table 3). This was the case for ASD (35, 63), cognitive functioning (58, 61), and PTSD (48, 64). The group that aimed at ASD-related symptoms such as lowered ability of recognition skills (35, 63) was shown to have a moderate non-significant (*P* > 0.05) effect size [*g* = 0.46 (95% CI 0.10–0.81, NNT = 3.91)]. The group targeting cognitive functioning-related symptoms such as lower attentional ability (58, 61) showed a large significant effect [*g* = 0.79 (95% CI 0.36–1.21, *P* < 0.05, NNT = 2.36)]. The group targeting PTSD-related symptoms such as flashbacks (48, 64) showed a moderate significant effect size [*g* = 0.59 (95% CI 0.20–0.99, *P* < 0.05, NNT = 3.09)].

We also conducted two subgroup analyses, grouping the studies by age and participant type. The group targeting youth (≤18) (34, 35, 59–61, 63) was shown to have a moderate effect size [*g* = 0.70 (95% CI 0.32–1.07, NNT = 2.63)] differing non-significantly from the adult group (18+) (48, 58, 64) [*g* = 0.53 (95% CI 0.07–0.99, NNT = 3.42)]. The group targeting clinical participants (with diagnosed mental disorder) (35, 59, 61, 63) showed a moderate effect size of [*g* = 0.59 (95% CI 0.18–0.99, NNT = 3.09)] differing non-significantly from the non-clinical group (34, 48, 58, 64) [*g* = 0.68 (95% CI 0.26–1.09, NNT = 2.70)].

## Discussion

### Summary of Results

This study aimed to give an overview of serious games for mental health-related symptoms that were evaluated with RCTs.

There were eight different games in our study. One of the games, SPARX, is both a goal-oriented and problem-solving game. The games Guardian Angel and JDTP are goal-oriented games. Five games (Tetris, Let’s Face It!, Braingame Brian, Lumosity, and “path-finding”) were categorized as cognition training games. In order to ascertain which game genre works best in targeting specific mental disorder symptoms more exploration is needed and studies need to be replicated.

All of the serious games were PC applications that required no internet connection to play. However, trends show that PC sales are declining, tablet sales are increasing, more time is spent on the smartphone and that most time spent on the smartphone is in playing games (in the US). It seems that the development and/or validation of serious games for mental health on this technical platform is lagging behind. As Fleming et al. (83) remarked, many mental health apps are already available. It is clear that considerable opportunities lie in this area. Accessibility and feasibility can be improved if this area is utilized. Software can be conveniently downloaded or distributed in app stores already available on smartphones.

We included 10 studies in the review that comprised 8 different serious games. The studies targeted depression-related symptoms, ASD, PTSD, ADHD, cognitive functioning, and AUD. The results of the meta-analysis showed a mean moderate effect size of these serious games for reducing psychiatric disorder-related symptoms. This finding is similar to that of a recently conducted meta-analysis of game-based interventions by Li et al. (36) for depression only. The effect sizes found in this study correspond to NNTs of approximately 3, indicating that three patients have to be treated in order to have one who would benefit, slightly lower than Li and colleagues. However, Li and colleagues did not confine their meta-analysis to serious games—they included simulation studies as well. The clinical impact for the youth group (≤18, *g* = 0.70, *n* = 5) is comparable to the adult group (*g* = 0.53), as in the Li et al.’s study (36).

These findings indicate that serious games for mental health-related symptoms have potential for various age groups. Our results compare favorably to those found in the review of serious games for depression only by Fleming et al. (83). This review was not confined to RCTs. Comparing our results with internet interventions that are not serious game based is more complex due to the differences in setting, study designs, and outcomes applied. A very generic comparison, with similar results could be made with the meta-analysis of Ebert et al. (27) on youth (2015) who likewise found evidence for the moderate efficacy of internet and computer-based CBT in the treatment of depression and anxiety (*g* = 0.76). Regarding adults, another meta-analysis on the impact of internet-based depression interventions (18 RCTs) of Cowpertwait and Clarke (84) likewise showed that web-based interventions targeting adult depression reduced symptoms moderately significantly compared to controls (*g* = 0.43). This study also showed that reminders and guidance are important moderators for treatment outcome.

The majority of studies focused on youth and young adults especially for ASD, ADHD, and depression (34, 35, 59–61, 63). Games for children with ASD-related symptoms showed comparable moderate effect as found by Grynszpan et al. (85) for technology-based (non-serious gaming) interventions for children with autism spectrum-related symptoms (*d* = 0.47). Studies using serious games targeting cognitive functioning were available for both adults and youth.

No serious games primarily targeting anxiety were found in this study. This finding may not be surprising, because this can be expected of a relatively new field of research. Another explanation could be that game elements do not add much more to VRET, which is found to have potential to combat anxiety (86–88).

### Strengths and Limitations

The strength of this study is that it is the first to provide insight into the potential effectiveness of serious games on psychiatric disorder-related symptoms based on RCTs only and on a strict definition of what is regarded to be a “serious game.” This is important as RCTs are considered the gold standard of research to measure effectiveness of interventions and to make clear what a serious game is or is not. This study has some limitations as well. First, the number of studies that were eligible for inclusion in this review was small and the number of participants included in those studies likewise. The risk of bias in these studies was unclear in many cases due to incomplete reporting and may have been a problem (selection, performance, and detection bias) indicating that the methodological quality of studies could be improved and effect sizes could be overestimated. Furthermore, given the small number of studies, several mental health disorders and clinical outcomes were collated as psychiatric-related disorders instead of focusing on single ones. This means the findings can only indicate improvement instead of clear symptom reduction (89). Such an approach is, however, not uncommon in an evolving new domain of academic endeavor [see, e.g., Ref. (89)]. As a consequence, we decided not to conduct subgroup analyses of distinct mental disorders as the number of studies and power for doing so was too low.

### Conclusion

Using serious games as interventions for reducing mental health problems appears feasible. Due to the limited number of RCTs that we have been able to include in this analysis, this review can only give an idea of the potential of serious games for treating mental disorders in the future. More RCTs are needed to determine the effectiveness of serious games. Future studies should not lose the technological development out of sight. Smartphone-based serious games for mental health need more exploration. Further, the effect and use of serious games for mental health that let players connect with other players using an internet connection need to be investigated.

## Author Contributions

HL: substantial contribution to every aspect of the manuscript, critical revision, and final approval and agrees to be accountable. JS: substantial contribution to design/conception, analysis, critical revision, and final approval and agrees to be accountable. TF: substantial contribution to interpretation of data, critical revision, and final approval and agrees to be accountable. HR: substantial contribution to design/conception, analysis, interpretation of data, critical revision, and final approval and agrees to be accountable.

## Conflict of Interest Statement

TF is a codeveloper of SPARX computerized therapy for depression and can benefit from any commercialization of it outside of New Zealand. The remaining authors have no conflict of interest to declare.
